# The Future of Medicinal Chemistry, PROTAC, and Undruggable Drug Targets

**DOI:** 10.1021/acs.jmedchem.1c01126

**Published:** 2021-07-23

**Authors:** Antti Poso

**Affiliations:** School of Pharmacy, University of Eastern Finland, P.O. BOX 1627, 70211 Kuopio, Finland; Department of Internal Medicine VIII, University Hospital Tübingen, Otfried-Müller-Strasse 14, 72076 Tübingen, Germany

## Abstract

WRD5 is a promising target for anticancer drug discovery. In addition, it plays a vital role in epigenetic regulation. Since biological inactivation of WRD5 is difficult to reach via classical approach, PROTACs (Proteolysis Targeting Chimeras) are offering a new option. In a study, published in this journal, new WRD5 targeting PROTACS are introduced. These new compounds, which are also active in cells, make it possible to evaluate the value of WRD5 as a drug target.

Drug design and especially anticancer drug design is extremely challenging field of science. In general, the aim is to interfere with some biochemical reactions or pathways by targeting human proteins with small molecules. Although classical enzyme inhibitors (like kinase inhibitors) or receptor ligands are useful, far too often the most interesting target proteins are beyond the possibilities of classical ligand design. This is especially true with many “undruggable” proteins like KRAS and MYC. Both proteins have been under intensive drug development work, but progress has been extremely slow. In the case of KRAS, the main issue has been the natural ligand GTP, its high affinity against KRAS, and extremely high endogenous ligand concentration within the cell. To compete with GTP, any drug molecule should have a picomolar-level IC_50_ value and should reach millimolar concentrations in cell. Naturally, these values are beyond reach, and thus, alternative approaches have been used. In the case of KRAS, the issue has been solved by a combination of structural biology and molecular modeling, resulting in the first FDA approved covalent KRAS inhibitor sotorasib.^1^ Despite this breakthrough, we are still far away from the situation that all critical proteins can be targeted. As an example, the KRAS inhibitor sotorasib is only useful against the G12C mutant and most KRAS mutants are still undruggable.

Another main issue is manifested in our attempts to find inhibitors against transcription factors like MYC or individual proteins of larger complexes, like WRD5 of WRAD. In this case, we do not have any reason to believe that classical drug design would be a viable option. One of the biggest obstacles is the lack of a druggable binding site. Indirect target inhibition, via protein–protein interaction inhibitors, is one realistic alternative. However, even this approach still has a critical question: how does one ensure biologically relevant downregulation of the target protein? PROTACs (Proteolysis Targeting Chimeras) are offering new possibilities in this task. Instead of blocking the biological function of the target protein by (non)covalent interactions, PROTACs promote protein degradation by matching the target protein with E3 ubiquitin ligases. This matching still requires selective and specific interaction (a ligand) against the target protein, but the true biological function is based on the proteolytic mechanism initiated by E3 ligase. The beauty of this approach is in the fact that any binding site almost anywhere on the protein surface, with a high enough binding affinity ligand, is a good starting point for PROTAC design. The site itself does not need to be biologically relevant, since biological function is based on the proteasomal degradation and not on the enzymatic inhibition or receptor inactivation.

Epigenetic regulation is one of those key biochemical functions which is difficult to target with normal ligands/inhibitors. In a study published in this this issue, Dölle et al. decided to target the so-called WRAD complex and especially WD repeat-containing protein 5 (WDR5). While WDR5 is an integral regulator for histone methyltransferases, it also directly interacts with MYC proteins. Interaction with class 2 lysine methyltransferases (KTM2) and with MYC occurs at the opposite binding regions. As KTM2 methyltransferases are critical in epigenetic regulation, WDR5 degradation would make it possible to target many oncogenic functions. At the same time, MYC proteins do require WDR5 for full activation, so WDR5 targeting is clearly a way to hit two major oncological targets simultaneously.

New WDR5 targeting PROTACS were designed based on existing WDR5 ligands, namely, OICR-9429 and pyrroloimidazole based inhibitors.^2^ As X-ray structures were available for both inhibitor classes, the optimal linker attachment point was easily detected (see Figure 1). Three different E3 ligase targeting ligands were used (CRBN, VHL, and MDM2), and linkers were based on PEG, aromatic, and aliphatic moieties. All together, 18 PROTACS were synthesized. Binding affinity was initially evaluated by DSF/temperature shift assay, and for selected compounds affinity and binding thermodynamics were assayed by ITC. The need for orthogonal assays is nicely demonstrated in this case, as many of the compounds with small thermal shifts still had binding affinity on the low two-digit nanomolar scale. Quite surprisingly, all eight compounds (according to ITC data) were very potent binders, ranging from 41 to 6 nM.

**Figure 1:**
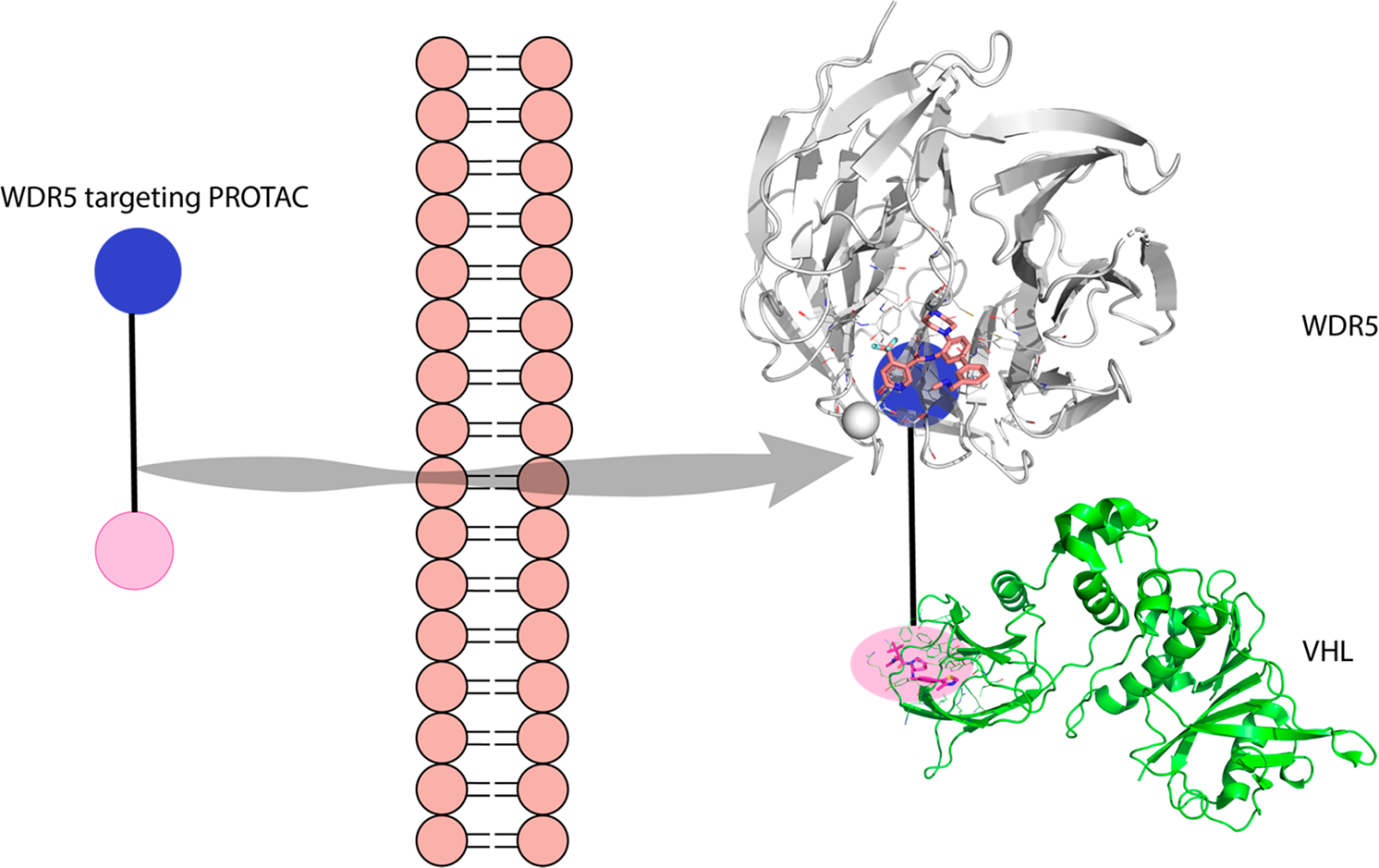
Schematic representation of the designed PROTACs. New compounds were able to cross the cell membrane and achieve biologically relevant WDR5 degradation. VHL structure 4W9H, and WDR5 structure 4QL1.

Naturally, cell permeability and target engagement in cells are also critical factors. This was validated by BRET assay, and indeed many of the studied compounds were shown to enter cells. However, MDM2 based compounds had weak permeation and those were not studied further. Both CRBN and VHL based compounds were permeable, but only VHL compounds resulted in functional WDR5 degradation in cells. Although the best compounds were active at the low nanomolar scale, only partial degradation was reached. An explanation for this is most probably the availability of target proteins since overexpression of VHL was able to increase PROTAC degradation effects. Thus, at the end, a functional PROTAC against WDR5 and activity in cells at the low nanomolar scale was reached.

Why these results are important? The short answer is clear: these compounds will serve as a proof that PROTACS do work against WDR5, and targeting difficult undruggable proteins is now closer to reality. In addition, inhibition of MYC by small molecules is getting more are more realistic option. It will be more than interesting to find out if these new compounds are also able to affect MYC pathways. If this is validated, scientists are able to design effective druglike molecules targeting WDR5 and MYC (although indirectly), and we should reconsider what “druggable” means. Personally, I would like to raise another point. The actual ligand or PROTAC design process was quite simple (although it required substantial background information and long research history). In this case, logical thinking was enough to create a new set of PROTACS. Although molecular modeling was used, it was mainly giving putative binding modes for already designed ligands. It is tempting to think that molecular modeling would be an effective tool for PROTAC-type ligand design, but we are still lacking fast and reliable enough protein–protein docking tools. In many cases PROTAC degradation efficiency is not directly related to ligand binding affinity but more on the ternary complex formation and stability. To model this, we must have much better molecular modeling tools.

The second point I would like to raise is simple. Should we all switch to PROTACS? Or do we still need classical medicinal chemistry? In fact, we need now more than ever medicinal chemistry, ligand design, and multidisciplinary work. Although PROTAC protein degradation efficiency is not directly related to protein binding affinity, one needs potent and selective binders for both ligases and target protein. At the same time, cell permeability, metabolic stability, and optimization of PROTAC ternary complex all are huge tasks. The work presented here is a nice example to demonstrate the future of medicinal chemistry.
